# [Retracted] Ubiquitin-specific protease 4 inhibits breast cancer cell growth through the upregulation of PDCD4

**DOI:** 10.3892/ijmm.2024.5417

**Published:** 2024-08-26

**Authors:** Yang Li, Daqing Jiang, Qi Zhang, Xiaoli Liu, Zhengang Cai

Int J Mol Med 38: 803-811, 2016; DOI: 10.3892/ijmm.2016.2685

Following the publication of this paper, it was drawn to the Editors' attention by a concerned reader that certain of the colony formation assay data shown in Fig. 4D on p. 807 and western blot assay data shown in Fig. 7A on p. 809 were strikingly similar to data appearing in different form other articles written by different authors at different research institutes that had already been published elsewhere prior to the submission of this paper to *International Journal of Molecular Medicine*. In view of the fact that the abovementioned data had already apparently been published previously, the Editor of *International Journal of Molecular Medicine* has decided that this paper should be retracted from the Journal. The authors were asked for an explanation to account for these concerns, but the Editorial Office did not receive a reply. The Editor apologizes to the readership for any inconvenience caused.

